# Improving repeatability of capillary electrophoresis—a critical comparison of ten different capillary inner surfaces and three criteria of peak identification

**DOI:** 10.1007/s00216-017-0382-y

**Published:** 2017-05-08

**Authors:** Paweł Mateusz Nowak, Michał Woźniakiewicz, Marta Gładysz, Magdalena Janus, Paweł Kościelniak

**Affiliations:** Faculty of Chemistry, Department of Analytical Chemistry, Jagiellonian University in Kraków, Ingardena St. 3, 30-060 Kraków, Poland

**Keywords:** Capillary electrophoresis, Capillary coating, Electrophoretic mobility, Micellar electrokinetic chromatography, Relative migration times, Repeatability

## Abstract

A poor repeatability of migration times caused by the fluctuations of electroosmotic flow (EOF) is an inherent weakness of capillary electrophoresis. Most researchers endeavor to prevent this problem using relative migration times or various capillary coatings which are expensive and not easy in comparison. Herein, we present an original approach to this problem: we apply a model sample designed to induce significant EOF instability, in order to critically compare ten capillary types with different physicochemical characteristics. Moreover, we accompany capillary modification with the evaluation of various criteria of peak identification: migration time, migration times ratio, and electrophoretic mobility. Our results show a great effectiveness of a dynamic coating in the stabilization of migration times, with the average RSD(%) value reduced from 3.5% (bare silica capillary) down to 0.5%. The good outcomes were also obtained for the surfactant-modified silica and amine capillaries. For the capillaries exhibiting significant instability of EOF, electrophoretic mobility turned out to be a more universal and reliable criterion of peak identification than relative migration time. It can be explained by an intrinsic dependency of migration times ratio on EOF change, which should always be considered during the selection of an internal standard.

**Electronic supplementary material:**

The online version of this article (doi:10.1007/s00216-017-0382-y) contains supplementary material, which is available to authorized users.

## Introduction

Capillary electrophoresis (CE) is one of the most powerful analytical techniques of a wide and still not fully uncovered analytical potential. It enables separation of the structurally similar molecules, including enantiomers, and provides low consumption of sample material and buffers, short analysis time, a variety of factors that can be easily used to improve resolution, and high automation degree. Nevertheless, CE has its own Achilles’ heels which often overshadow its huge analytical power—relatively poor repeatability/reproducibility and sensitivity [1–4]. The former becomes a significant limitation when the RSD values obtained for migration times surpass the acceptable limits, often over 3%. The alterations of migration velocity may be caused by many factors: fluctuations of electroosmotic flow (EOF), unstable temperature, current, pH, ionic strength, presence of air bubbles, and siphoning effect [1–4]. EOF fluctuations often pose the main source of this instability. They result from the physical modification of capillary inner surface, caused most commonly by the analyte-wall interactions, formation of insoluble aggregates, and insufficient capillary rinsing. Insofar as capillary regeneration may be enhanced by using the longer rinsing times and the additional solvents, the dynamical analyte-wall interactions occurring during the electromigration constitute a bigger problem and often hamper analysis. It is particularly frequently observed for the real samples with complex matrix and the protein-containing samples [5]. The large repeatedly ionized biomolecules possess many potential sites of such interactions. In such conditions, an average capillary lifetime is also appreciably reduced.

The most popular method for improving repeatability and for enabling transfer of methods in the qualitative analysis is the use of relative migration times, i.e., a ratio of migration times obtained for the analyte and the internal standard. Assuming an efficient temperature control, relative migration times do not depend on capillary dimensions, separation potential, and viscosity change. However, what can be overlooked by the less experienced users of the CE technique, effectiveness of relative migration times in reducing the EOF fluctuation-related error is by definition limited and depends strictly on a difference in mobility between analyte and internal standard (see the next part of manuscript for more details). An alternative way is to determine electrophoretic mobility of the analytes instead of migration times ratio or to perform the transformation of the whole electropherogram from a time scale into a mobility scale. Effectiveness of both methods has been examined and confirmed in many studies [6–19]; nevertheless, electrophoretic mobility is still quite rarely used as a direct criterion of peak identification.

Another approach to this problem is the application of physicochemically modified capillaries, coated permanently or dynamically with chemically versatile agents. The literature concerning this subject is broad, and among numerous articles, one may encounter the applications of the commercially offered ready-to-use capillaries, kits for dynamic coating, and other coating materials synthetized ab initio [5, 20–37]. The major advantage is that a modified capillary surface exhibits weaker affinity to analytes, especially macromolecules, stabilizes EOF, and thus allows one to enhance repeatability. Moreover, the use of polyamine positively ionized coating enables the EOF reversal, from cathodic (toward cathode) to anodic (toward anode), while the neutral capillaries provide its total elimination. On the other hand, the use of any type of coating significantly elevates the cost of analysis. The direct comparisons of various types of capillary modifications are rarely performed [27, 29, 33]. On that account, there is still little known about advantages and predispositions of the given capillary types in regard to the particular types of methods and experimental conditions, and this hinders their critical evaluation and selection.

This work offers a fresh look at the repeatability of the CE-based analyses affected by a variable EOF magnitude. We present an original approach, the use of a specially designed model sample inducing the significant flow instability and containing chemically varied analytes, combined with the concurrent examination of ten various capillary types of the different inner surfaces, and the comparison of three alternative criteria of peak identification: migration times, relative migration times, and electrophoretic mobilities. Our results may be of importance for all CE users and for researchers without experience in CE who look for an alternative technique for liquid and gas chromatography. The presented data help one to choose the optimal capillary type, and show that a careful consideration of many factors is crucial to select the optimal criterion of peak identification.

## Materials and methods

### Instrumentation

**Fig. 1:**
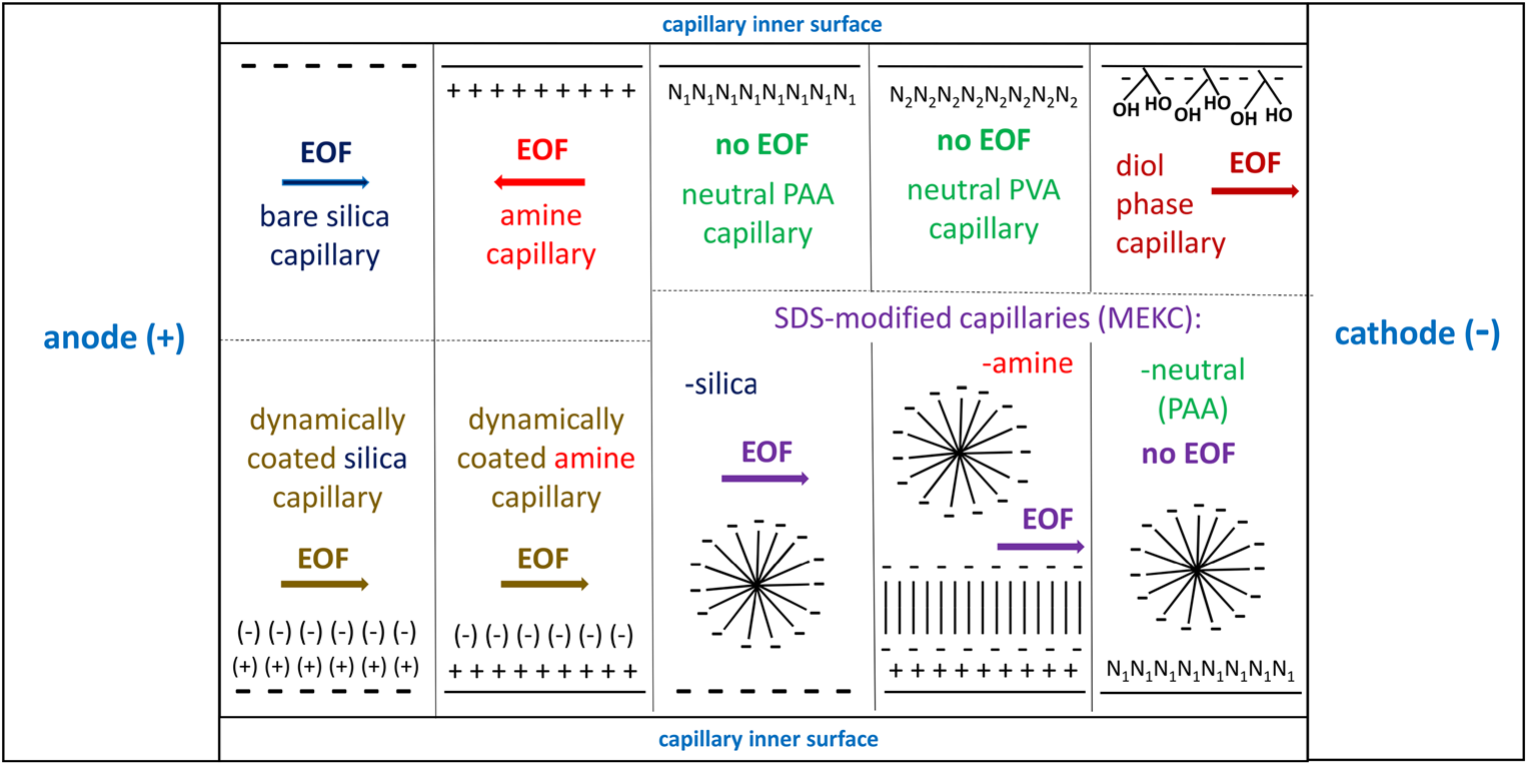
Schematic illustration of ten physicochemically different capillaries including their inner surface and the direction of EOF. Symbols: - negative charge; + positive charge; ( ) dynamic polyionic layer (renewable); N_1_ polyacrylamide (PAA) layer; N_2_ polyvinyl alcohol (PVA) layer; II hydrophobic tail-tail interactions. MEKC-SDS method is presented as a distinct capillary modification (see the text)

The experiments were performed with a P/ACE MDQ Capillary Electrophoresis (CE) System (Beckman-Coulter, Brea, CA, USA) equipped with a diode array detector. The following commercially available capillaries were applied: an uncoated bare fused-silica capillary (silica); amine eCAP™ polyamine-coated capillary (amine) providing the reversal of EOF; neutral polyacrylamide-coated capillary (neutral PAA): and neutral polyvinyl alcohol-coated capillary (neutral PVA) providing neutralization of EOF, all supplied by Beckman-Coulter, and a Celerity™ diol phase-coated capillary column (diol) supplied by MicroSolve Technology, Eatontown, NJ, USA. All capillaries were of 60-cm total length, 50-cm effective length, and 50-μm internal diameter. In order to obtain other physicochemical modifications, the silica and amine capillaries were subjected to a dynamic coating performed with the commercially available kit CEofix™ pH 6, containing the initiator™ and accelerator™ solutions (Beckman-Coulter): obtaining the CEofix™ dynamically coated bare silica capillary (DC-silica) and CEofix™ dynamically coated amine eCAP™ capillary (DC-amine). Coating by a successive multiple ionic layer (SMIL) was performed with the silica capillary (cationic layer was formed by initiator™ and anionic layer was formed by accelerator™), and by a single anionic-polymer layer with the amine capillary, pre-coated permanently by a polyamine layer. In addition, in the case of bare silica, amine, and neutral PAA capillaries, the background electrolyte (BGE) was modified by addition of 30 mM sodium dodecyl sulfate (SDS). Since the presence of micelles may influence on all dynamic analyte-wall interactions, these variants were included in the comparison as the independent physicochemical modifications. In the case of amine capillary, SDS molecules coat the capillary’s wall forming a characteristic admicelle layer, reversing its charge [38]. Schematic illustration of all ten capillary types is shown in Fig. 1.

The sample trays and capillaries were conditioned at 25 °C. The rinsing of capillaries between the runs was done by applying a pressure of 137.9 kPa (20 psi). The procedures were either developed in our laboratory or followed the instructions provided by the suppliers. They are presented in detail in Table 1. BGE was composed of the phosphate buffer (Na_2_HPO_4_/NaH_2_PO_4_) of the 50 mM ionic strength and pH 6.0, except the dynamic coating where BGE (accelerator) was supplied as a kit component and was not prepared ab initio in the laboratory. It was also the phosphate buffer; its pH was equilibrated before use to 6.0. Its ionic strength was unknown, but probably similar, as it follows from the comparison of current measured during separations. BGE based on the phosphate buffer was selected to provide the same conditions for all capillaries, including the commercial kit (CEofix™). All aqueous solutions were prepared using a deionized water (Milli-Q system, Merck-Millipore, Darmstadt, Germany), filtered through a 0.45-μm regenerated cellulose membrane and then degassed by sonication and centrifugation. The separation voltage of 20 kV was always applied. The reverse polarity (cathode at inlet) was used in the case of amine, neutral, and SDS-modified neutral capillaries, while the normal polarity (anode at inlet) in all other cases. The current values were between 45 and 60 μA for all capillary types. To avoid significant increase of separation time, the positive pressure of 2.8 kPa (0.4 psi) was used in case of the neutral capillaries (without SDS) where EOF is virtually entirely eliminated. Sample injection was performed using the pressure of 2.8 kPa (0.4 psi) for 4.0 s. The UV-vis absorption spectra were collected between 200 and 600 nm; 200 nm was the analytical wavelength for plotting electropherograms. The analysis of electropherograms was done using the Origin 9.1. software (OriginLab Corporation, Northampton, MA, USA).

**Table 1:** The procedures used for capillary rinsing, before the first use on a given working day and between the following runs

Full capillary name/(abbreviation)	Before the first use^a^	Between runs
Uncoated bare fused-silica capillary/(silica)	Methanol: 20 min 0.1 M HCl: 5 min Deionized H_2_O: 5 min 0.1 M NaOH: 20 min BGE: 20 min	0.1 M NaOH: 3 min BGE: 3 min
Amine eCAP™ polyamine-coated capillary/(amine)	Deionized H_2_O: 20 min Amine regenerator solution (supplied): 20 min BGE: 20 min	Amine regenerator solution (supplied): 3 min BGE: 3 min
Neutral polyacrylamide-coated capillary/(neutral PAA)	Deionized H_2_O: 20 min BGE: 20 min	Deionized H_2_O: 3 min BGE: 3 min
Neutral polyvinyl alcohol-coated capillary/(neutral PVA)	Deionized H_2_O: 20 min BGE: 20 min	Deionized H_2_O: 3 min BGE: 3 min
Celerity™ diol phase-coated capillary/(diol)	Deionized H_2_O: 20 min 0.1 M HCl: 5 min Deionized H_2_O: 5 min 0.1 M NaOH: 20 min BGE: 20 min	0.1 M NaOH: 3 min BGE: 3 min
CEofix™ dynamically coated bare silica capillary/(DC-silica)	Methanol: 20 min 0.1 M HCl: 5 min Deionized H_2_O: 5 min 0.1 M NaOH: 20 min BGE (normal): 20 min	0.1 M NaOH: 3 min Initiator—poly-cation (supplied): 3 min BGE—with accelerator (supplied): 3 min
CEofix™ dynamically coated amine eCAP™ capillary/(DC-amine)	Deionized H_2_O: 20 min Amine regenerator solution (supplied): 20 min BGE (normal): 20 min	Amine regenerator solution (supplied): 3 min BGE—with accelerator (supplied): 3 min
SDS-modified bare silica capillary/(SDS-silica)	Methanol: 20 min 0.1 M HCl: 5 min Deionized H_2_O: 5 min 0.1 M NaOH: 20 min BGE—with SDS: 20 min	0.1 M NaOH: 3 min BGE—with SDS: 3 min
SDS-modified amine eCAP™ capillary/(SDS-amine)	Deionized H_2_O: 20 min Amine regenerator solution (supplied): 20 min BGE—with SDS: 20 min	Amine regenerator solution (supplied): 3 min BGE—with SDS: 3 min
SDS-modified neutral polyacrylamide-coated capillary/(SDS-neutral (PAA))	Deionized H_2_O: 20 min BGE—with SDS: 20 min	Deionized H_2_O: 3 min BGE—with SDS: 3 min

^a^For the fresh capillary conditioning, the duration of each individual step was doubled

### Sample composition

The sample composition was selected taking several issues into account: (i) the presence of macromolecule intrinsically prone to interactions with capillary inner surface; (ii) the presence of small molecules of different charge, to include analytes migrating both before and after EOF marker; (iii) the presence of neutral EOF marker; and (iv) simplicity, to avoid potential problems with peaks overlapping, especially in the surfactant-modified capillaries. The final composition was as follows: human serum albumin (HSA), molecular weight (MW)≈66,500 Da—macromolecule known for its nonspecific adsorption/adhesion to silica surface via various types of interactions, positively charged amitriptyline (AMI), MW = 277 Da, p*K*_a_≈9.4, negatively charged warfarin (WAR), MW = 308 Da, p*K*_a_≈5.0 [39], and EOF marker—dimethyl sulfoxide (DMSO), MW = 78 Da, all dissolved in BGE. In the case of DC-silica, DC-amine, and SDS-modified capillaries, the standard BGE without kit components and buffer additives was used for sample preparation. All analytes were supplied by Sigma-Aldrich (St. Louis, MO, USA). Three concentration levels of each analyte were used: 500, 250, and 125 μg mL^−1^. To keep the sample composition as simple as possible, only one macromolecule was used, but instead, relatively high concentration range was applied. The repetitions number amounted to 6 for each concentration level. The new vials with fresh BGE solutions were used for each concentration level, to minimize buffer depletion.

### Calculations

The values of electrophoretic mobility (*μ*_ep_) were obtained from Eq.1:

1 $$ {\mu}_{\mathrm{ep}}=\frac{L_{\mathrm{tot}}\bullet {L}_{\mathrm{eff}}}{V}\bullet \left(\frac{1}{t_{\mathrm{tot}}}-\frac{1}{t_{\mathrm{eof}}}\right) $$

where *L*_tot_ and *L*_eff_ are the total and effective capillary lengths (cm), *V* is the nominal separation voltage set up in the software (kV); *t*_tot_ is the total (absolute) migration time of analyte (min); while *t*_eof_ is the time measured for the neutral marker of EOF—DMSO (min).

## Results and discussion

### Theoretical background

**Fig. 2:**
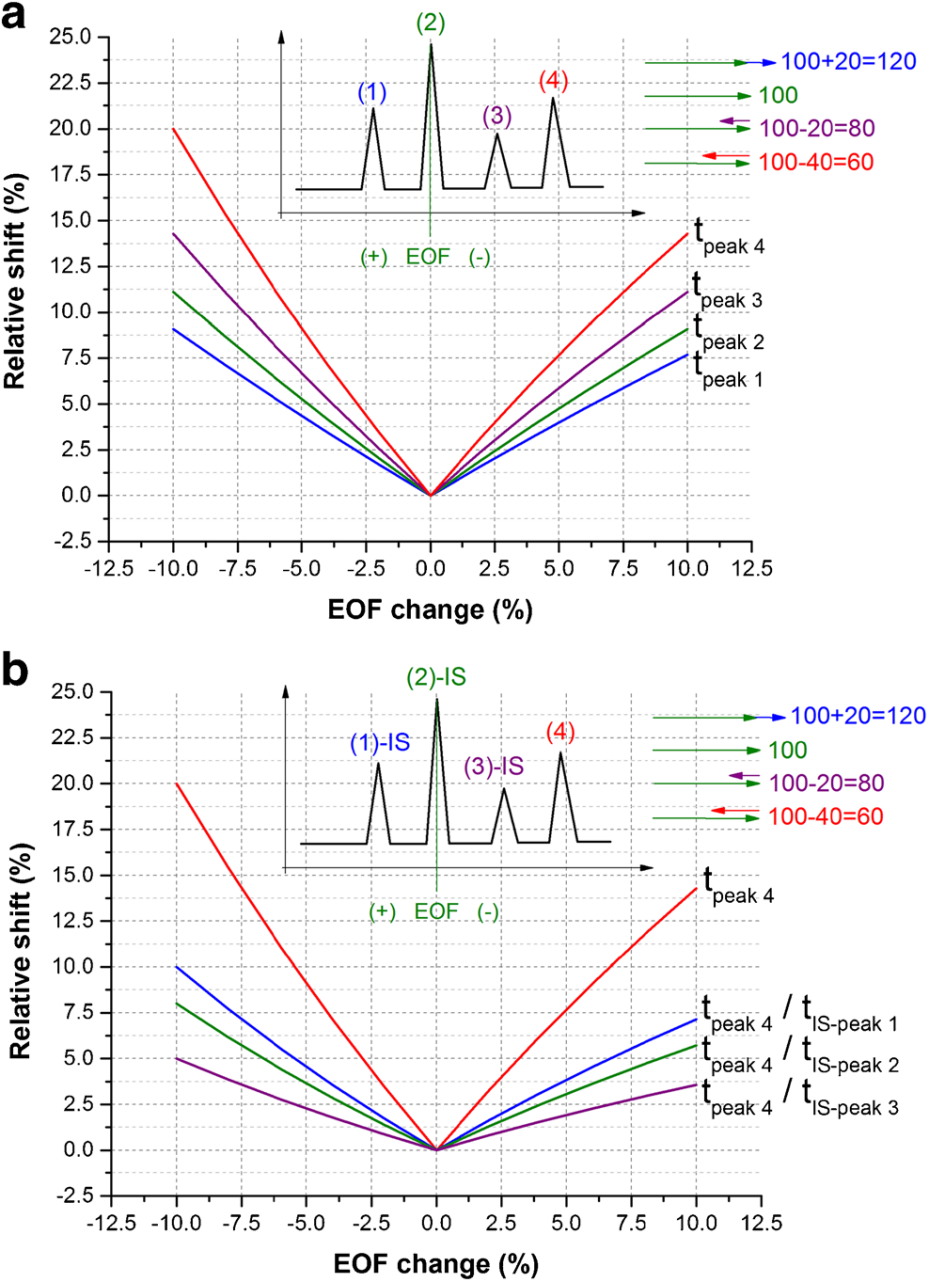
Theoretical simulation of the potential shifts of parameters in the qualitative analysis caused by the EOF change, **(A**) Using migration times obtained for four different compounds (1–4) exhibiting different migration velocity. **(B**) Using migration time and relative migration times obtained for one compound (4) and considering three different internal standards (IS, 1–3). The shifts were calculated as a relative change of the parameter upon the given EOF alteration. The *inset graphics* present schematic electropherogram and adding of vectors for the particular analytes (the initial electroosmotic mobility equals 100 and it varies +/− 10; electrophoretic mobilities are constant and they equal +20, 0, −20, −40 for the given analytes; the values were chosen arbitrarily to visualize the discussed phenomenon)

In CE, migration times are inversely proportional to the apparent mobility, which derives directly from the sum of two vectors: electrophoretic and electroosmotic ones. It is to be noted that the change of EOF affects only the electroosmotic vector and entails disproportionate shifts of migration times for different peaks. It is shown in Fig. 2A, where we consider the separation of four distinct model analytes with the conventional bare silica capillary. The same change in the EOF vector induces different relative changes in the total vector for the given analytes. Therefore, there is no proportionality in the shift of migration times; the smallest change is observed for the fastest compound (cationic), and the largest one for the slowest compound (second anionic). In this respect, the analytes of a large negative electrophoretic mobility are inherently much more susceptible to EOF variation than the cationic compounds.

It is also seen that due to the non-proportional shift of migration times, the relative migration times are also changing, and their utilization only partially eliminates the EOF change-related error (Fig. 2B). The more similar is mobility of analyte and standard; the lower error can be expected. It would be entirely eliminated then, and only then, if internal standard had the same migration time as analyte.

### Repeatability of migration times

**Fig. 3:**
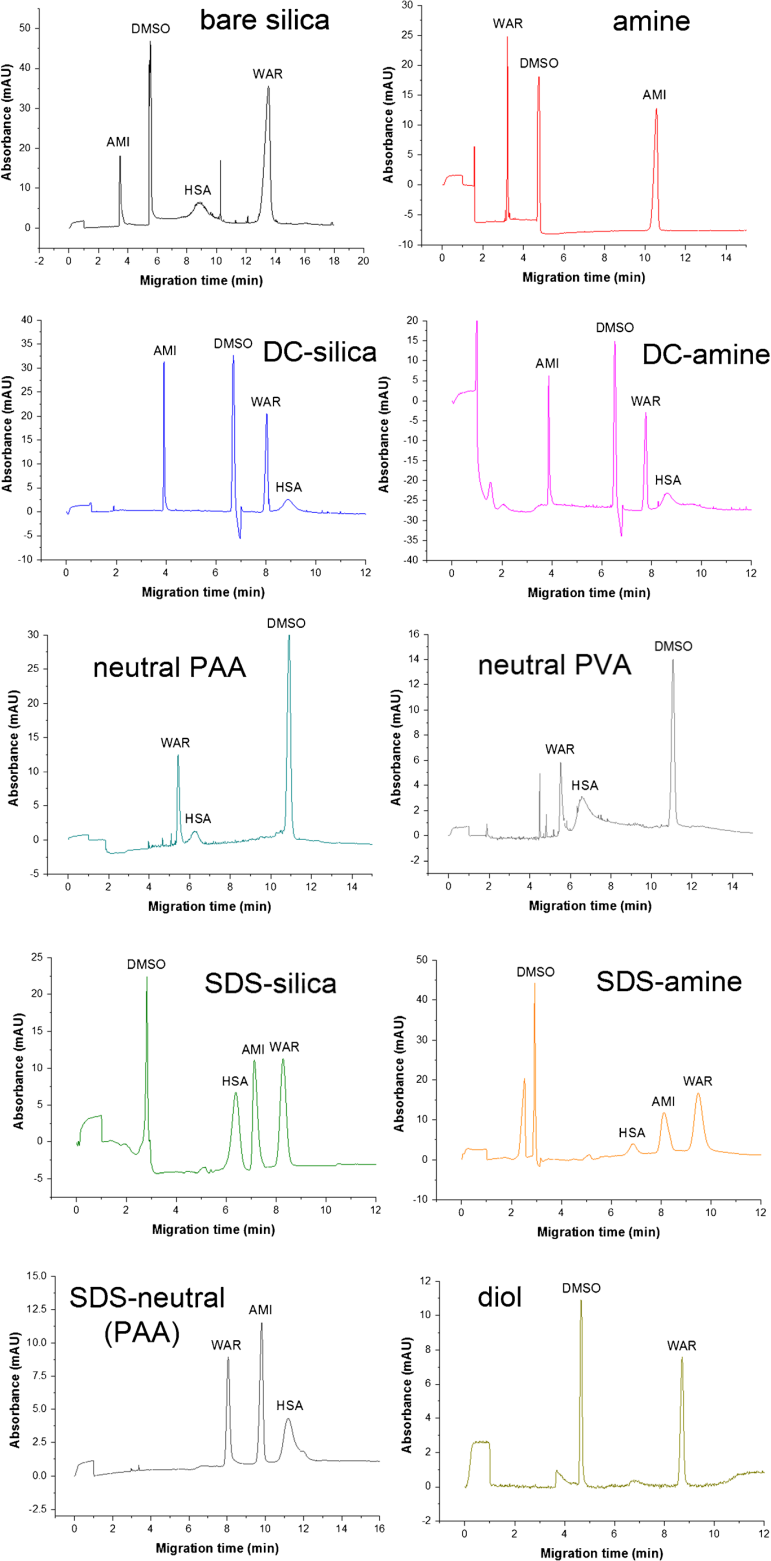
Representative electropherograms obtained for all ten capillaries on the highest concentration level (500 μg × mL^−1^). The negative peaks observed for the DC-silica, DC-amine and SDS-amine capillaries may stem from the lack of kit components and SDS molecules in the sample solution. The EOF strength was measured using always the positive DMSO peak

After performing separation of the model sample using ten different capillary types, the repeatability of migration times was expressed by the RSD values, depicted in Table 2. The representative electropherograms are shown in Fig. 3, while the values of migration times are shown in Table S1 in the Electronic Supplementary Material (ESM). The first issue is the effect of capillary clogging, which was noted for the several independent bare silica capillaries. Every time the capillary got clogged after around 8–12 separations, making further analysis is impossible. It was observed as the appearance of artifacts on the electropherogram, significant drift of the baseline and the lack of the signal from analytes. This effect was not observed with the other capillaries, suggesting that the interaction between HSA and “unprotected” silanol groups may be the main obstacle hampering analysis. A similar effect was observed by us in our recent work, when HSA was used as an p*K*_a_-shift inducer [33].

**Table 2:** The RSD (%) values (*n* = 6) obtained for various analytes on three concentration (conc.) levels (500, 250, 125 μg × mL^−1^) using ten different capillaries, and calculated for three alternative parameters: *t*—migration times, *t/t*_IS_—relative migration times (calculated in respect to DMSO as internal standard), *μ*_ep_—electrophoretic mobilities

Parameter	Analyte	Conc.	Silica	Amine	DC-silica	DC-amine	Neutral PAA	Neutral PVA	SDS-silica	SDS-amine	SDS-neutral (PAA)	Diol
*t*	AMI	500	1.2	6.2	0.2	0.0	X	X	11.4	8.4	2.3	X
		250	C	5.9	0.0	1.0	X	X	0.3	3.7	1.2	X
		125	C	6.3	0.2	4.1	X	X	0.2	0.9	3.7	X
	WAR	500	5.9	1.2	0.4	0.5	1.1	0.8	10.3	5.5	2.9	7.3
		250	C	1.3	0.1	1.6	1.5	1.5	0.3	3.8	2.8	8.4
		125	C	1.2	0.2	5.2	1.1	0.9	0.4	0.6	4.4	8.2
	DMSO	500	2.2	2.5	0.3	0.3	0.2	0.1	3.4	2.5	X	3.1
		250	C	2.5	0.2	1.4	3.2	2.9	0.4	0.9	X	2.6
		125	C	2.3	0.2	4.8	0.8	1.3	0.0	0.4	X	3.6
	HSA	500	4.9	X	1.2	0.6	0.8	4.2	8.6	5.6	1.6	X
		250	C	X	0.3	2.7	1.0	1.9	0.8	3.2	0.7	X
		125	C	X	0.8	3.3	1.9	1.7	0.1	0.7	2.7	X
*t*_*/*_ *t*_IS_	AMI	500	1.1	4.0	0.2	0.3	X	X	7.7	6.0	X	X
		250	C	3.4	0.2	0.6	X	X	0.2	2.9	X	X
		125	C	4.0	0.1	0.9	X	X	0.2	0.7	X	X
	WAR	500	3.8	1.3	0.2	0.2	1.1	0.8	6.6	3.1	X	4.2
		250	C	1.2	0.2	0.3	1.8	1.6	0.2	3.0	X	6.0
		125	C	1.2	0.2	0.4	0.4	0.6	0.4	0.6	X	4.7
	HSA	500	1.6	X	1.0	0.4	0.7	4.3	5.0	3.2	X	X
		250	C	X	0.3	1.3	0.6	1.1	0.6	2.4	X	X
		125	C	X	0.7	1.8	0.4	0.6	0.1	0.7	X	X
*μ*_ep_	AMI	500	1.3	2.0	0.3	0.4	X	X	0.4	0.3	X	X
		250	C	0.2	0.2	0.7	X	X	0.6	0.6	X	X
		125	C	0.1	0.3	2.9	X	X	0.1	0.4	X	X
	WAR	500	0.8	1.7	1.0	1.0	2.2	1.6	0.7	1.0	X	1.3
		250	C	1.3	1.0	0.9	0.2	0.9	0.6	1.1	X	1.9
		125	C	1.2	0.8	2.2	1.4	0.2	0.3	0.5	X	0.8
	HSA	500	2.3	X	2.7	0.9	1.8	4.9	0.5	0.6	X	X
		250	C	X	0.9	2.8	0.8	1.5	0.7	1.1	X	X
		125	C	X	1.8	1.2	1.3	1.4	0.1	0.7	X	X

The capillary name abbreviations are consistent with Table 1; C—capillary clogging effect, X—lack of the corresponding peak due to adsorption of analyte on capillary inner surface or very low apparent mobility

The bare silica capillary (before clogging), two dynamically coated capillaries, and silica and amine SDS-modified capillaries enabled the detection of all sample ingredients (Fig. 3). The HSA peak was not observed with the amine capillary, most likely because of the strong protein-wall interactions caused by the positive charge of the wall and the negative net charge of the macromolecule. On the other hand, AMI was not detected using the neutral capillaries since the mechanically induced flow of buffer was too weak to register a cationic compound upon application of the reverse polarity. DMSO peak was not observed with the SDS-modified neutral capillary owing to the lack of EOF. The diol-modified capillary, in turn, exhibited most probably a strong retention of HSA and AMI, yielding only the peaks of DMSO and WAR. In this context, regarding the clogging of the bare silica capillary, the dynamically coated and SDS-modified silica and amine capillaries seem the best option for performing analysis of the chemically varied samples containing macromolecules as well as cationic and anionic compounds.

As regards the RSD values obtained for migration times, an interesting relation is observed for the bare silica and amine capillaries. In the former case, the RSD values rise in the order AMI > DMSO > HSA > WAR, the same as the migration order observed with this capillary. In the case of the amine capillary, WAR > DMSO > AMI order is noted, also consistent with the increase of migration times. This observation fully agrees with the expectation that due to the increasing EOF fluctuation-related error, discussed previously, repeatability of migration times should drop in accordance with migration order.

The dynamically coated silica capillary is characterized by the very low RSD values, averagely 0.78% for HSA and 0.20% for the rest of compounds, respectively. This excellent outcome is not preserved by the dynamically coated amine capillary, for which the RSD values start to increase from the second concentration level (250 μg × mL^−1^), with the increasing injections number. In this case, the positively charged polyionic layer is permanently present on the wall, unlike the dynamically coated silica capillary; hence, the HSA molecules are probably being retained on this layer from run to run and a specific “surface passivation effect” takes place. As it comes to the neutral capillaries, the RSD values are moderate and no clear trend is visible. Nevertheless, the neutral PAA capillary yields better results than the neutral PVA capillary. The SDS-modified silica and amine capillaries are characterized by the high RSD values on the first concentration level (500 μg × mL^−1^), but on the second and third levels (250 and 125 μg × mL^−1^) applied chronologically later, the RSD values become significantly reduced. This is an opposite effect to the dynamically coated amine capillary where the higher RSD values were observed on the second and third concentration levels; here, a specific surface stabilization and “maturation” may occur as a result of the increasing injection number. Importantly, the high RSD values noted on the first concentration level also agree with the effect discussed in the previous section. In this separation medium, electrophoretic mobility of all the analytes, except DMSO, is negative and significant due to the interactions with the negatively ionized SDS molecules. For this reason, the RSD values obtained for AMI, WAR, and HSA are much higher than those obtained for DMSO. The SDS-modified neutral capillary displays a different characteristic. The RSD values are not changing along the sequence of runs, and they are rather moderate. Finally, the diol-modified capillary yields a weak repeatability, comparable for all three concentration levels and, again, significantly worse for the anionic WAR detected long after DMSO.

In conclusion, for this type of sample, the dynamic coating performed with the silica capillary is appreciably better than any other capillary type. In reference to the other published works [33, 36, 37], the effectiveness of the amine- and diol-modified capillaries in comparison to the bare silica capillary occurred to be noticeably worse. The dynamically coated silica capillary, contrary to the previous observations [37], turned out to be more effective in the stabilization of migration times than the dynamically coated amine capillary. This effect seems to be specific for the sample containing HSA molecules possessing the negative net charge.

### Other criteria of peak identification

As it is seen in Table 2, the repeatability of relative migration times is in general better than the absolute times, irrespective of the analyte and capillary type. This confirms the well-documented observations that a ratio of migration times is less sensitive to EOF change than absolute migration time, independent of the analyte charge and size [2, 3, 6, 7]. However, the differences observed in case of the dynamically coated silica and both neutral capillaries are small, and they should be regarded as statistically insignificant. The comparison of the RSD values obtained for relative migration times using the various internal standards is shown in Table 3. This comparison includes all possible combinations, to present how repeatability of data is correlated with the difference in electrophoretic mobility and the nature of internal standard.

**Table 3:** The RSD(%) values averaged from three concentration levels (500, 250, 125 μg × mL^−1^) obtained for the relative migration times using different capillaries and calculated for various internal standards (IS)

Analyte	IS	silica	Amine	DC-silica	DC-amine	Neutral PAA	Neutral PVA	SDS-silica	SDS-amine	Diol
AMI	WAR	4.7	5.0	0.2	0.8	–	–	0.6	1.5	–
	DMSO	1.1	3.8	0.2	0.6	–	–	2.7	3.2	–
	HSA	5.2	–	0.7	1.2	–	–	1.2	1.4	–
WAR	AMI	4.7	5.0	0.2	0.8	–	–	0.6	1.5	–
	DMSO	3.8	1.3	0.2	0.3	1.1	1.0	2.4	2.2	5.0
	HSA	8.8	–	0.6	1.2	0.8	1.9	0.8	0.7	–
DMSO	AMI	1.1	3.8	0.2	0.6	–	–	2.5	3.2	–
	WAR	3.8	1.3	0.2	0.3	1.1	1.0	2.2	2.2	5.1
	HSA	5.8	–	0.7	1.1	1.2	2.1	1.8	2.1	–
HSA	AMI	5.4	–	0.7	1.2	–	–	1.1	1.4	–
	WAR	9.5	–	0.6	1.2	0.8	1.9	0.8	0.7	–
	DMSO	1.6	–	0.7	1.1	1.2	2.0	1.9	2.1	–

The capillary name abbreviations are consistent with Table 1

Taking into account four possible internal standards, the differences observed for the dynamically coated and neutral capillaries are low, within the margin of the random error. In the other capillaries, almost in each case the worst repeatability and the highest RSD values are observed for HSA used as an internal standard. The RSD value obtained for WAR as an analyte and HSA as a standard in the bare silica capillary reaches even 8.8%. It can be explained by the nonspecific protein-wall interactions, leading to the adsorption of macromolecule and the significant peak broadening. Regarding AMI, WAR, and DMSO as possible internal standards, the differences are noticeable for the bare silica-, amine-, and both SDS-modified capillaries. There is a tendency that the more similar the mobilities of the analyte and the internal standard are, the lower RSD is noted. For example, DMSO is a better standard than WAR for the identification of the AMI peak in the bare silica and amine capillaries, whereas much worse in the SDS-modified capillaries where WAR and AMI migrate with a similar velocity (see electropherograms in Fig. 3). This confirms the theoretical divagations presented previously.

Another issue is the comparison between relative migration times and electrophoretic mobilities (see Table 2 for the RSD values and Table S1 in ESM for the calculated mobilities). It is interesting than when we consider the highest RSD values obtained for the absolute migration times in this experiment, those over 5%, the use of electrophoretic mobility allows to improve repeatability much more effectively than the use of relative migration time calculated in respect to DMSO (i.e., using the same input value as in the case of electrophoretic mobility where DMSO plays a role of the EOF marker). This effect is visible for WAR identified with the bare silica- and diol-modified capillaries, AMI in case of the amine capillary, and for all compounds in case of the SDS-modified capillaries. Interestingly, the consistent observation was also reported in our recent work, when we used the uncoated capillary and the sample devoid of protein [40]. However, as it was mentioned, in the SDS-modified capillaries, both AMI and WAR (detected within a comparable time) are better standards than DMSO. In consequence, some RSD values obtained for relative migration times with different standards are lower, similar to those obtained for electrophoretic mobilities (compare Tables 2 and 3). This is, however, not observed for the bare silica and amine capillaries; there, the application of other standards than DMSO deteriorates the repeatability. These effects stem from the intrinsic dependency of relative migration times on EOF magnitude, which can be minimized by selecting the internal standard of a similar mobility as the analyte (see section “Theoretical background”).

It should also be pointed out that the repeatability of absolute migration times, relative migration times, and electrophoretic mobilities may be very similar, as in the case of AMI detected with the bare silica capillary, WAR detected with the amine capillary, or all molecules detected with the dynamically coated silica capillary. In this situation, the influence of EOF instability is low, and the other random errors are decisive. Most importantly, the repeatability of relative migration times and electrophoretic mobilities may be decreased by the fact that two migration times are required to calculate these parameters, obtained for the analyte and the standard/marker, respectively. This introduces the increased random uncertainty as compared to the absolute migration times, stemming, e.g., from peak broadening and its asymmetry, instability of current and temperature during separation, and analyte-wall interactions [2, 6]. Therefore, a thorough consideration of all these factors is crucial for the selection of an appropriate criterion of peak identification.

### Electroosmotic mobility

**Fig. 4:**
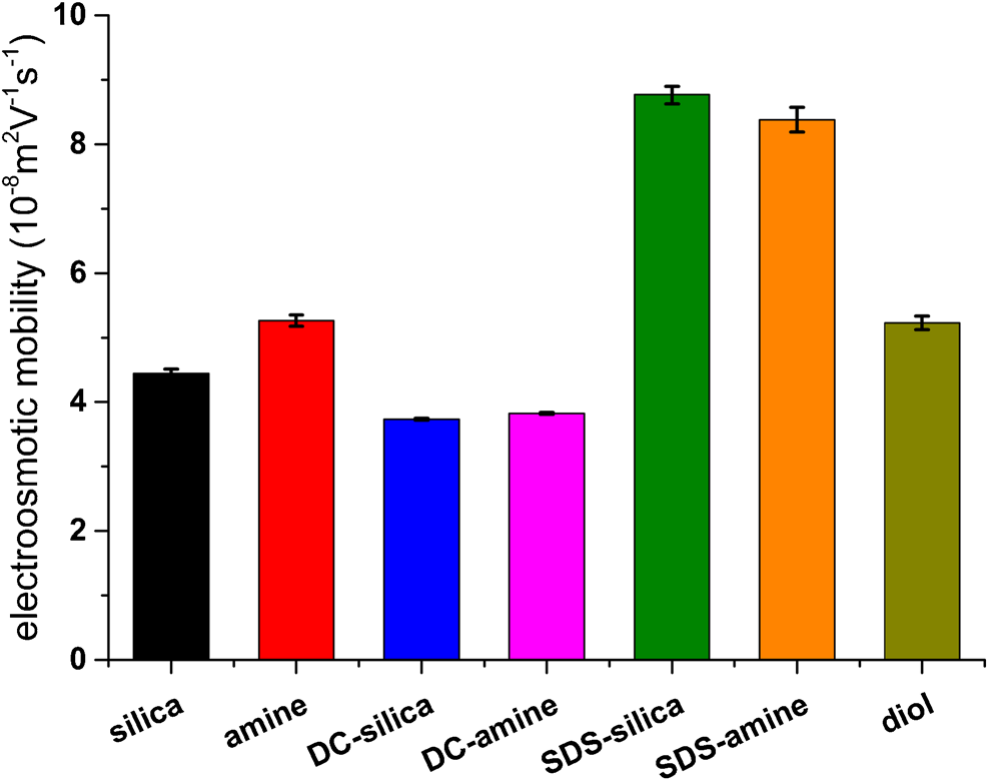
The average values of electroosmotic mobility obtained for various capillaries, using the sample containing all analytes on the highest analyte concentration level (500 μg × mL^−1^)

The magnitude of EOF was calculated for each capillary and presented in Fig. 4. The significant difference in comparison to the bare silica capillary is observed only for the SDS-modified capillaries. Nevertheless, due to the interactions with the negatively ionized SDS micelles, the overall separation time is actually similar as in the other capillaries. It is also interesting that in the present experimental conditions, the amine capillary exhibits the similar electroosmotic mobility as the uncoated capillary, unlike the separations performed in the different buffer systems [35]. This outcome confirms that when the amine capillary is applied with the phosphate buffer, EOF is reduced due to the specific interaction between the positively charged wall and the phosphate buffer components [37, 41]. Therefore, aside from the other benefits, all modified capillaries tested in this experiment are rather useless in decreasing a total separation time in this experimental setup.

## Conclusions

A detrimental impact of the analyte-wall interactions on the capillary lifetime and the repeatability of migration times may be successfully prevented by the capillary coating or addition of surfactant to BGE. The dynamic coating (CEofix™) allows one to appreciably reduce the EOF fluctuations for the chemically varied and protein-rich samples. Noticeably, in this case, the use of electrophoretic mobilities and relative migration times instead of absolute times does not bring an improvement because of the prevalent impact of other random errors, increased by the use of standard/marker. The application of the dynamic poly-cationic layer (initiator solution™) is crucial for preserving functionality of coating; its replacement by the permanently present amine layer (eCAP amine capillary™) may have an unfavorable impact on the repeatability. After stabilization/pre-treatment of the inner surface, the SDS-modified silica and amine capillaries are also effective in the stabilization of migration times. The remaining capillaries are evidently more prone to the analyte-wall interactions; however, any modified capillary allows one to avoid capillary clogging, which in this study, was inevitable with the bare silica capillary. When the variation of absolute migration times is significant, over 2–3%, the use of relative migration times or electrophoretic mobilities is advised to ensure the reliable peak identification. In the former case, it is crucial to choose the internal standard of a similar mobility to the analyte. It is caused by the fact that relative migration times are intrinsically dependent on EOF change, although they are less sensitive than absolute times. Therefore, electrophoretic mobilities seem to be a more universal and reliable criterion of peak identification when a large EOF variation is expectable, or when the mobility of the analytes differs considerably. Nevertheless, a suitable correction of the inherent systematic errors attributed to the mobility determination is required to provide the high reliability and broad applicability, in particular the Joule heating-related effects [42]. The reliable electrophoretic mobility values may be useful in performing method transfer between different experimental setups, e.g., capillary types, dimensions, or separation voltages. The presented data may be helpful in development of versatile CE-based analytical and bioanalytical methods and in the critical comparison of CE with other separation techniques. They show some effective strategies how to the overcome the inherent weakness of CE.

## Electronic supplementary material

Table S1 (PDF 116 kb)

## Abbreviations

AMI: Amitriptyline
BGE: Background electrolyte
CE: Capillary electrophoresis
DC: Dynamic coating
EOF: Electroosmotic flow
HSA: Human serum albumin
MEKC: Micellar electrokinetic chromatography
SDS: Sodium dodecyl sulfate
WAR: Warfarin
