# Supplementary material for: Improving repeatability of capillary electrophoresis—a critical comparison of ten different capillary inner surfaces and three criteria of peak identification
Source: Anal Bioanal Chem. 2017 May 8;409(18):4383–93. doi: 10.1007/s00216-017-0382-y (PMC5486911; doi:10.1007/s00216-017-0382-y)
Supplement: Supplementary file 1 — (PDF 116 kb) [file 216_2017_382_MOESM1_ESM.pdf]

Analytical and Bioanalytical Chemistry

Electronic Supplementary Material

Improving repeatability of capillary electrophoresis – a critical comparison of ten different capillary inner surfaces and three criteria of peak identification

Paweł Mateusz Nowak, Michał Woźniakiewicz, Marta Gładysz, Magdalena Janus,
Paweł Kościelniak

Table S1 The values of absolute migration times – t (min), electrophoretic mobilities – μ_{ep} ($10^{-8}m^2V^{-1}s^{-1}$) and electroosmotic mobilities – μ_{eof} ($10^{-8}m^2V^{-1}s^{-1}$), measured in ten capillaries of the different inner surface

parameter	*analyte*	*conc.*	**silica**	**amine**	**DC-silica**	**DC-amine**	**neutral PAA**	**neutral PVA**	**SDS-silica**	**SDS-amine**	**SDS-neutral (PAA)**	**diol**
		**500**	3.51	10.56	3.91	3.87	-	-	7.39	8.68	9.33	-
	**AMI**	**250**	-	11.28	3.94	3.98	-	-	6.74	8.34	9.20	-
		**125**	-	14.78	3.94	4.18	-	-	6.77	8.45	9.32	-
		**500**	14.78	3.20	8.05	7.81	5.50	5.46	8.57	10.40	7.84	9.28
	**WAR**	**250**	-	3.28	8.12	8.10	5.62	5.53	7.60	9.96	7.84	10.48
		**125**	-	3.52	8.13	8.58	5.51	5.51	7.66	10.02	8.00	13.74
**t**		**500**	5.62	4.75	6.70	6.54	10.92	11.07	2.85	2.98	-	4.78
	**DMSO**	**250**	-	4.89	6.76	6.78	11.61	11.63	2.90	2.95	-	5.07
		**125**	-	5.44	6.77	7.17	10.97	11.65	2.74	2.95	-	5.55
		**500**	9.68		8.99	8.70	6.35	6.34	6.54	7.33	10.77	-
	**HSA**	**250**	-		9.14	9.25	6.52	6.51	5.99	7.08	10.77	-
		**125**	-		9.19	9.74	6.56	6.45	5.98	7.17	11.06	-
		**500**	1.79	-1.93	1.78	1.76	-	-	-3.57	-3.66	-	-
	**AMI**	**250**	-	-1.93	1.76	1.73	-	-	-3.27	-3.65	-	-
		**125**	-	-1.93	1.76	1.64	-	-	-3.62	-3.68	-	-
		**500**	-1.83	1.69	-0.42	-0.42	1.50	1.55	-3.88	-3.98	-	1.69
**μ_{ep}**	**WAR**	**250**	-	1.68	-0.41	-0.40	1.56	1.57	-3.56	-3.97	-	1.79
		**125**	-	1.67	-0.41	-0.38	1.52	1.60	-3.91	-3.99	-	1.69
		**500**	-1.19	-	-0.63	-0.63	1.10	1.08	-3.28	-3.31	-	-
	**HSA**	**250**	-	-	-0.64	-0.66	1.13	1.11	-2.96	-3.30	-	-
		**125**	-	-	-0.65	-0.63	1.05	1.17	-3.30	-3.33	-	-
		**500**	4.45	5.27	3.73	3.82	2.29	2.26	8.76	8.38	-	5.23
**μ_{eof}**	**DMSO**	**250**	-	5.11	3.70	3.69	2.15	2.15	8.62	8.47	-	4.93
		**125**	-	4.60	3.69	3.49	2.28	2.15	9.12	8.48	-	4.50

500, 250, 125 – concentration (conc.) levels (µg·mL^{-1}). The values of migration times ratios can be easily obtained using the absolute migration times values presented in the table.
